# High production of 2,3-butanediol from biodiesel-derived crude glycerol by metabolically engineered *Klebsiella oxytoca* M1

**DOI:** 10.1186/s13068-015-0336-6

**Published:** 2015-09-15

**Authors:** Sukhyeong Cho, Taeyeon Kim, Han Min Woo, Yunje Kim, Jinwon Lee, Youngsoon Um

**Affiliations:** Clean Energy Research Center, Korea Institute of Science and Technology (KIST), Hwarangno 14-gil 5, Seongbuk-gu, Seoul, 136-791 Republic of Korea; Interdisciplinary Program in Agriculture Biotechnology, Collage of Agriculture and Life Science, Seoul National University, 1 Gwanak-ro, Gwanak-gu, Seoul, Republic of Korea; Clean Energy and Chemical Engineering, Korea University of Science and Technology, 217 Gajeong-ro, Yuseong-gu, Daejeon, Republic of Korea; Department of Chemical and Biomolecular Engineering, Sogang University, 35 Baekbeom-ro, Mapo-gu, Seoul, Republic of Korea

**Keywords:** 2,3-butanediol, *Klebsiella oxytoca*, Biodiesel-derived crude glycerol, Fermentation

## Abstract

**Background:**

2,3-Butanediol (2,3-BDO) is a promising bio-based chemical because of its wide industrial applications. Previous studies on microbial production of 2,3-BDO has focused on sugar fermentation. Alternatively, biodiesel-derived crude glycerol can be used as a cheap resource for 2,3-BDO production; however, a considerable formation of 1,3-propanediol (1,3-PDO) and low concentration, productivity, and yield of 2,3-BDO from glycerol fermentation are limitations.

**Results:**

Here, we report a high production of 2,3-BDO from crude glycerol using the engineered *Klebsiella oxytoca* M3 in which *pduC* (encoding glycerol dehydratase large subunit) and *ldhA* (encoding lactate dehydrogenase) were deleted to reduce the formation of 1,3-PDO and lactic acid. In fed-batch fermentation with the parent strain *K. oxytoca* M1, crude glycerol was more effective than pure glycerol as a carbon source in 2,3-BDO production (59.4 vs. 73.8 g/L) and by-product reduction (1,3-PDO, 8.9 vs. 3.7 g/L; lactic acid, 18.6 vs. 9.8 g/L). When the double mutant was used in fed-batch fermentation with pure glycerol, cell growth and glycerol consumption were significantly enhanced and 2,3-BDO production was 1.9-fold higher than that of the parent strain (59.4 vs. 115.0 g/L) with 6.9 g/L of 1,3-PDO and a small amount of lactic acid (0.7 g/L). Notably, when crude glycerol was supplied, the double mutant showed 1,3-PDO-free 2,3-BDO production with high concentration (131.5 g/L), productivity (0.84 g/L/h), and yield (0.44 g/g crude glycerol). This result is the highest 2,3-BDO production from glycerol fermentation to date.

**Conclusions:**

2,3-BDO production from glycerol was dramatically enhanced by disruption of the *pduC* and *ldhA* genes in *K. oxytoca* M1 and 1,3-PDO-free 2,3-BDO production was achieved by using the double mutant and crude glycerol. 2,3-BDO production obtained in this study is comparable to 2,3-BDO production from sugar fermentation, demonstrating the feasibility of economic industrial 2,3-BDO production using crude glycerol.

**Electronic supplementary material:**

The online version of this article (doi:10.1186/s13068-015-0336-6) contains supplementary material, which is available to authorized users.

## Background

In the last few years, considerable effort and progress have been made in the production of bio-based bulk chemicals from renewable resources because of the decrease in fossil fuel availability and increasing concern for global warming [1]. 2,3-Butanediol (2,3-BDO) is a promising bio-based bulk chemical due to numerous industrial applications, such as the manufacture of printing inks, perfumes, softening and moistening agents, pharmaceuticals, anti-freeze agent, and liquid fuels [2, 3]. In addition, methyl ethyl ketone (an organic solvent for resins and lacquers) and 1,3-butadiene (a monomer for synthetic rubber) can be produced by the hydration of 2,3-BDO [3, 4].

Previous studies on biological production of 2,3-BDO have focused on sugar fermentation using glucose [5–10] and sucrose [11] as the carbon sources. Because of the relatively high cost of conventional sugars, 2,3-BDO production has been investigated using a non-edible inexpensive lignocellulosic biomass and organic waste, such as corncobs [12], corn stover [13], Jerusalem artichoke tubers [14], *Jatropha* hulls [15], and sugarcane molasses [16].

**Fig. 1:**
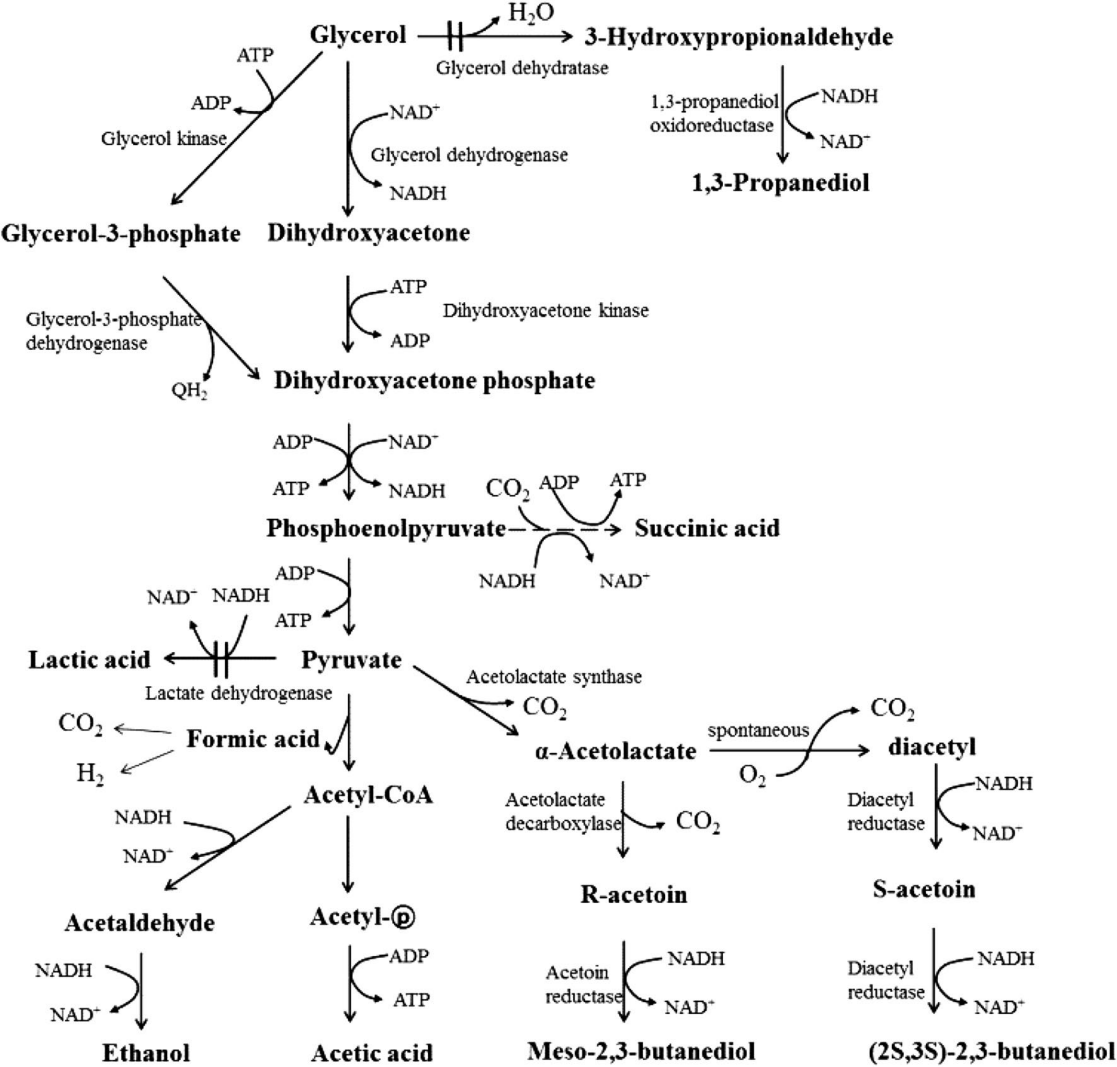
Metabolic pathway of 2,3-BDO from glycerol in *K. oxytoca* M1. The *crossed double line* is the pathway deleted in this study

Glycerol, which is generated as a by-product from ethanol fermentation, fat saponification, and biodiesel production [17], is also an attractive cheap resource for 2,3-BDO production. In particular, because the amount of biodiesel-derived crude glycerol is almost equivalent to 10 % (w/w) of global biodiesel production, there is an increasing surplus of glycerol on the world market [18]. *Klebsiella* species, such as *K. pneumoniae* and *K. oxytoca,* have been found to utilize glycerol as the sole carbon source and produce 1,3-propanediol (1,3-PDO) and 2,3-BDO [18–22]. Conversion of glycerol to 2,3-BDO occurs through the oxidative pathway, where glycerol is converted to dihydroxyacetone phosphate (DHAP) via glycerol-3-phosphate in the presence of electron acceptors (e.g., O_2_ under aerobic conditions) or via dihydroxyacetone in the absence of oxygen (i.e., fermentative route) (Fig. 1) [23]. In addition to the oxidative branch, glycerol is also metabolized through the reductive pathway, which results in the generation of 1,3-PDO (Fig. 1). 1,3-PDO is a major by-product generated during the production of 2,3-BDO using glycerol and may serve as an obstacle for obtaining a high purity of 2,3-BDO in downstream processes because of the similar boiling points of 2,3-BDO and 1,3-PDO [3].

Several reports have investigated the effect of dissolved oxygen level [24] and pH [20, 21] on 2,3-BDO production and the ratio of 1,3-PDO:2,3-BDO using glycerol as the sole carbon source. Until now, Petrov and Petrova [21] have reported the highest 2,3-BDO production (70 g/L) from pure glycerol by *K. pneumoniae* at the productivity of 0.47 g/L/h through forced pH fluctuation (consecutive raising of pH with ΔpH = 1 at every 12 h using 5 N sodium hydroxide); however, 1,3-PDO was still generated up to 16.3 g/L, and this result was obtained using pure glycerol, not crude glycero1. Yang et al. [25] demonstrated 1,3-PDO-free 2,3-BDO production (83.3 and 0.87 g/L/h) using *Bacillus amyloliquefaciens* by supplying beet molasses as a co-substrate in addition to biodiesel-derived crude glycerol; however, when biodiesel-derived glycerol was supplied as the sole carbon source, much lower 2,3-BDO concentration (43.1 g/L) and productivity (0.45 g/L/h) were obtained by *B. amyloliquefaciens.* Moreover, considering that the 2,3-BDO titer and productivity levels obtained from glucose fermentation by *K. pneumoniae* (101.5–150.0 g/L and 2.54–4.21 g/L/h) [5, 7] and *K. oxytoca* (113–130 g/L and 1.63–2.1 g/L/h) [6, 8] were much higher than those obtained using glycerol as the sole carbon source, much improvement in 2,3-BDO production from crude glycerol is required to facilitate a cost-effective high 2,3-BDO production.

Previously, a newly isolated *K. oxytoca* M1 was reported to be able to produce 2,3-BDO and acetoin selectively as the main products depending on temperature [26]. In this study, we attempted a high 2,3-BDO production using biodiesel-derived crude glycerol as the sole carbon source by the metabolically engineered *K. oxytoca* M3, in which *pduC* (encoding glycerol dehydratase large subunit, accession number AIE72369) and *ldhA* (encoding lactate dehydrogenase, accession number AIE70186) were deleted to reduce the formation of by-products, such as 1,3-PDO and lactic acid. The double deletion mutant showed a significantly improved 2,3-BDO production from pure glycerol and crude glycerol in fed-batch fermentation compared to the parent strain. Notably, 1,3-PDO-free 2,3-BDO production by the double deletion mutant was observed when crude glycerol was used as the carbon source in fed-batch fermentation. To our knowledge, the final titer (131.5 g/L), the productivity (0.84 g/L/h), and the yield (0.44 g/g crude glycerol) of 2,3-BDO from crude glycerol obtained by the double deletion mutant were the highest recorded in 2,3-BDO production from glycerol as the sole carbon source to date.

## Results and discussion

### Flask fermentation of *K. oxytoca* M1 using pure and crude glycerol

To evaluate glycerol utilization and 2,3-BDO production by *K. oxytoca* M1, flask batch fermentation was performed with pure glycerol and crude glycerol as the sole carbon sources at the initial concentration of 35–40 g/L. As shown in Table 1, the performance of the 2,3-BDO production by *K. oxytoca* M1 was similar regardless of the type of glycerol (Table 1). Cell growth (dry cell weight, DCW) appeared to be inhibited by crude glycerol. Several components in crude glycerol, such as free fatty acid, methanol, MONG (matter organic non-glycerol), and salt, are known to cause cell growth inhibition [27, 28]. However, considering that the final pH of crude glycerol fermentation was lower than the pH value of pure glycerol fermentation (pH 4.9 vs pH 6.0), a lower DCW in crude glycerol fermentation might be caused by a low pH level as presented in the next section. Overall, *K. oxytoca* M1 could produce 2,3-BDO as the main product using glycerol. Notably, 1,3-PDO, which is known to be one of the main by-products in 2,3-BDO fermentation by *Klebsiella* species [19–21], was not detected for *K. oxytoca* M1 in flask fermentation. Therefore, *K. oxytoca* M1 was further investigated as a potential strain for 2,3-BDO production from glycerol.

**Table 1:** Comparison of flask batch fermentations by *K. oxytoca* M1 using pure and crude glycerol

	Pure glycerol	Crude glycerol
Glycerol consumption (g/L)	33.9 ± 0.27	37.8 ± 0.74
Dry cell weight (g/L)	2.2 ± 0.29	1.6 ± 0.10
Lactic acid (g/L)	0.3 ± 0.59	4.2 ± 0.13
Ethanol (g/L)	2.5 ± 0.39	3.1 ± 0.13
1,3-PDO (g/L)	0	0
2,3-BDO (g/L)	8.4 ± 0.46	8.9 ± 0.26
2,3-BDO productivity (g/L/H)	0.17 ± 0.01	0.19 ± 0.01
2,3-BDO yield (g/g)	0.25 ± 0.02	0.24 ± 0.00
Final pH	6.0 ± 0.04	4.9 ± 0.01
Time (h)	48	48

The data are given as average ± standard deviation of triplicate experiments

### Fed-batch fermentations of *K. oxytoca* M1 using pure and crude glycerol

**Fig. 2:**
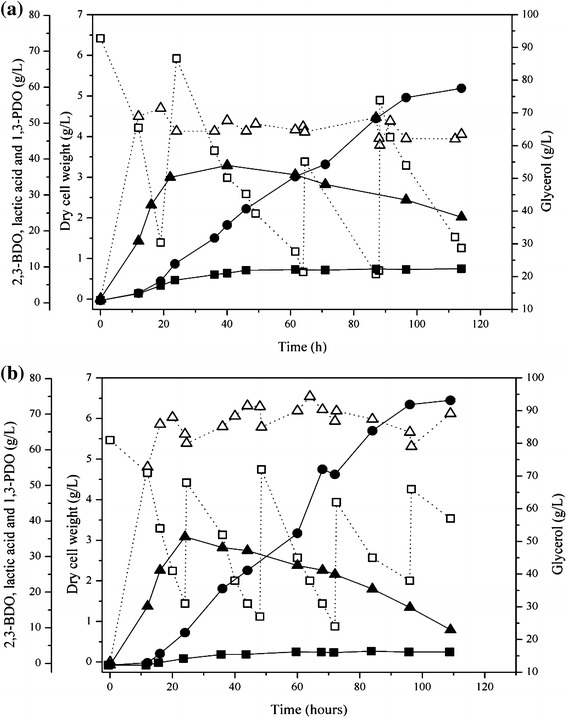
Time course of 2,3-BDO fermentation using pure glycerol and crude glycerol by *K. oxytoca* M1. **a** Fed-batch fermentation with pure glycerol. **b** Fed-batch fermentation with crude glycerol. The following symbols were used: dry cell weight (*unfilled* *triangle*), residual glycerol (*unfilled square*), 2,3-BDO (*filled circle*), 1,3-PDO (*filled square*), and lactic acid (*filled triangle*)

To investigate the feasibility of a high 2,3-BDO production by *K. oxytoca* M1 from glycerol, fed-batch fermentation using pure glycerol was conducted with pH control (pH = 6) in a 3 L-bioreactor. Fed-batch fermentation using crude glycerol was also performed to investigate whether the accumulation of impurities in crude glycerol would inhibit the cell growth and 2,3-BDO production of *K. oxytoca* M1. When fed-batch fermentation using pure glycerol was carried out for 114 h, *K. oxytoca* M1 successfully produced 2,3-BDO at concentrations up to 59.4 g/L with the productivity of 0.52 g/L/h (Table 2; Fig. 2a). Interestingly, when crude glycerol was used in fermentation, DCW (6.1 g/L) and 2,3-BDO concentrations (73.8 g/L) as well as 2,3-BDO productivity (0.68 g/L/h) were much higher than those achieved using pure glycerol (Table 2; Fig. 2b). Similarly, it has been reported that crude glycerol exhibited positive effects on glycerol consumption and 1,3-PDO production for *K. pneumoniae* [27]. Until now, 70 g/L has been the highest reported 2,3-BDO concentration from pure glycerol as the sole carbon source at a productivity of 0.47 g/L/h using forced pH fluctuations (ΔpH = 1 at every 12 h) [21]. In this study, higher concentration (73.8 g/L) and productivity (0.68 g/L/h) in comparison to previous results were obtained by simply maintaining the pH level at 6 and using crude glycerol.

**Table 2:** Comparison of fed-batch fermentations by *K. oxytoca* M1 and *K. oxytoca* M3 using pure and crude glycerol

Strain	*K. oxytoca* M1		*K. oxytoca* M3	
	Pure glycerol	Crude glycerol	Pure glycerol	Crude glycerol
Glycerol consumption (g/L)	189.5	177.7	297.1	300.3
Dry cell weight (g/L)	4.1	6.1	5.8	9.0
2,3-BDO (g/L)	59.4	73.8	115.0	131.5
1,3-PDO (g/L)	8.9	3.7	6.9	0
Lactic acid (g/L)	18.6	9.8	0.7	0.8
Ethanol (g/L)	4.0	1.9	9.3	1.7
2,3-BDO productivity (g/L/h)	0.52	0.68	1.01	0.84
2,3-BDO yield (g/g)	0.31	0.42	0.39	0.44
Fermentation time (h)	114	109	114	156

Lactic acid and 1,3-PDO productions during the fed-batch fermentation (Table 2) were different from those observed during flask fermentation (Table 1). Lactic acid production from pure glycerol rapidly increased to 30.5 g/L after 40 h and decreased to 18.6 g/L (Fig. 2a). The production of lactic acid from crude glycerol was higher than that with pure glycerol during 24 h of fermentation (35.7 g/L at 24 h), but decreased to 9.8 g/L at 109 h. In contrast to the flask fermentation results (Table 1), 1,3-PDO was produced in fed-batch fermentation from both pure and crude glycerol with concentrations up to 8.9 and 3.7 g/L, respectively (Table 2). Co-production of 1,3-PDO and lactic acid along with 2,3-BDO production has been generally observed in the fermentation of *Klebsiella* species using glycerol as the sole carbon source [20, 21, 29]. Previous studies have reported that the product distribution greatly depended on the pH level; 1,3-PDO and lactic acid formation tended to increase with pH control at 7, while 2,3-BDO production increased without pH control (final pH = 4.3–5.1) [20]. In the fed-batch fermentation of *K. oxytoca* M1, because the pH level was kept at 6, it might have caused the increase of 1,3-PDO and lactic acid formation in comparison with flask cultivation. When fed-batch fermentation of *K. oxytoca* M1 was conducted without pH control using pure glycerol, 2,3-BDO was produced mainly with a trace of 1,3-PDO and lactic acid; but, once pH was decreased below 4.9, glycerol was not utilized anymore at that point (data not shown). Thus, further fed-batch fermentation was conducted with pH control at 6.

### Construction of the *pduC* deletion mutant and batch fermentation of the mutant using pure glycerol

Although *K. oxytoca* M1 could produce 2,3-BDO using crude glycerol at a higher concentration and productivity levels than those achieved in previous studies, by-product formation needed to be decreased for efficient conversion of glycerol to 2,3-BDO. To reduce the formation of 1,3-PDO and lactic acid, the genes encoding glycerol dehydratase large subunit (PduC), which is responsible for the first step of 1,3-PDO synthesis from glycerol, and lactate dehydrogenase (LDH) converting pyruvate to lactic acid were chosen for deletion.

Even though blocking 1,3-PDO formation seems necessary for 2,3-BDO production from glycerol, there has been no study regarding the effect of the deletion of *pduC*, the gene encoding PduC, on cell growth and 2,3-BDO production. Thus, to investigate whether the deletion of *pduC* exhibited adverse effects on cell growth and 2,3-BDO production, *K. oxytoca* M1 *pduC* deletion mutant was first constructed using the λ Red recombination system [30]. PCR result and nucleotide sequencing data confirmed that the *pduC* gene of *K. oxytoca* M1 was successfully deleted (Figs. 1a) and this mutant strain was named *K. oxytoca* M2 (Table 3).

**Table 3:** Bacterial strains and plasmids used in this study

Strains or plasmids	Genotype and relevant characteristics	Source or references
Strains		
*K. oxytoca* M1	Parent strain	[26]
*K. oxytoca* M2	*K. oxytoca* M1 Δ*pduC*	This study
*K. oxytoca* M3	*K. oxytoca* M1 Δ*pduC*Δ*ldhA*	This study
*K. oxytoca* KCTC1686	Type strain	[33]
*E. coli* DH5α	*supE*44*, ΔlacU*169, (*φ80lac*ZΔM15), *hsdR*17, *recA*1, *endA*1, *gyr*A96, *thi*-1, *relA*1	Invitrogen
Plasmids		
pRedET	Derivative of pSC101, Tet^R^, temperature sensitive, carrying lambda red recombinase	Gene bridge
707-FLPe	Derivative of pSC101, Tet^R^, temperature sensitive, containing an FLPe recombinase	Gene bridge
pTOP-FCF	Derivative of pUC, containing an FRT-flanked Cm^R^ cassette-involved vector	[34]

**Fig. 3:**
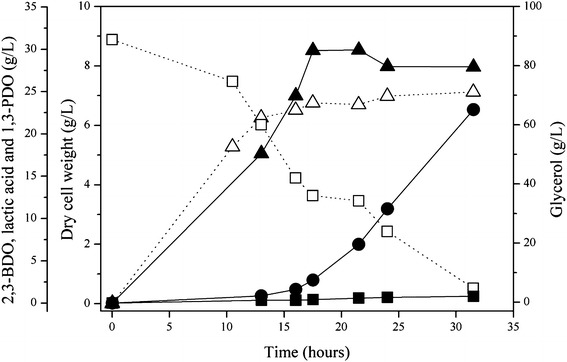
Time course of 2,3-BDO fermentation using pure glycerol by *K. oxytoca* M1 Δ*pduC*. The following symbols were used: dry cell weight (*unfilled* *triangle*), residual glycerol (*unfilled square*), 2,3-BDO (*filled circle*), 1,3-PDO (*filled square*), and lactic acid (*filled triangle*)

When batch fermentation was conducted with *K. oxytoca* M2 strain with the pH level maintained at 6.0 (Fig. 3), *pduC* deletion showed a positive effect on cell growth and 2,3-BDO production compared to the parent strain. Moreover, the deletion of *pduC* gene of *K. oxytoca* M1 resulted in nearly abolished 1,3-PDO formation (0.8 g/L of 1,3-PDO) in comparison with the 1,3-PDO production of the parent strain (7.2 g/L at 36 h in Fig. 2a). This demonstrates that the disruption of *pduC* was effective for the reduction of 1,3-PDO formation. However, lactic acid was still produced in concentrations up to 30 g/L at 21.5 h, and 2,3-BDO production was not significantly improved in comparison with the parent strain.

### Construction of *pduC* and *ldhA* double deletion mutant and fed-batch fermentation of the double mutant using pure glycerol

To reduce lactic acid production, we generated the double deletion mutant strain *K. oxytoca* M1 Δ*pduC*Δ*ldhA* (i.e., *K. oxytoca* M3) in which the *ldhA* gene was abrogated from the *pduC* mutant (i.e., *K. oxytoca* M2) (Table 3). Successful deletion of *ldhA* was confirmed by PCR amplification of the *ldhA* flank region with the primers ldhAcon1 and ldhAcon2 (steps 2 and 3 in Additional file 1: Fig. S1B) and nucleotide sequencing data. While the fragment of 1100 bp (Additional file 1: Fig. S2, lane 4) containing the intact *ldhA* gene was amplified from the parent strain, the fragment of 150 bp (Additional file 1: Fig. S2, lane 5) was identified in the mutant strain using the primers ldhAcon1 and ldhAcon2. This mutant strain was named *K. oxytoca* M3 (Table 3).

**Fig. 4:**
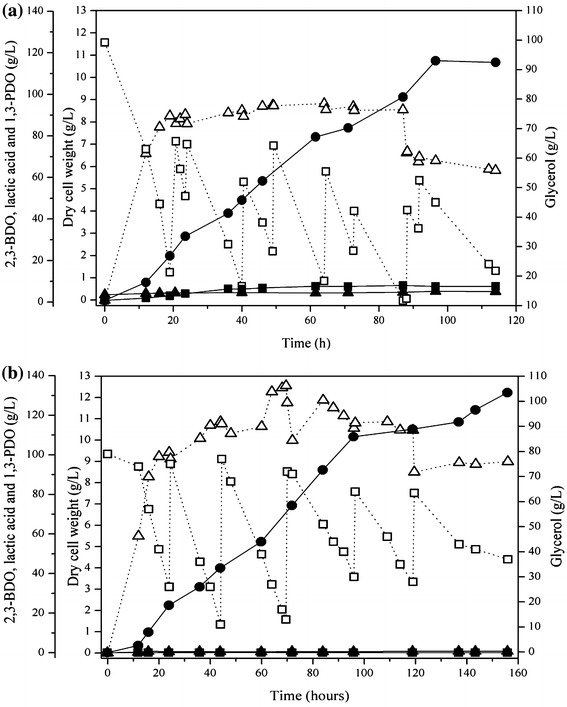
Time course of 2,3-BDO fermentation using pure glycerol and crude glycerol by *K. oxytoca* M1 Δ*pduC*Δ*ldhA*. **a** Fed-batch fermentation with pure glycerol. **b** Fed-batch fermentation with crude glycerol. The following symbols were used: dry cell weight *unfilled* *triangle*, residual glycerol *unfilled square*, 2,3-BDO *filled circle*, 1,3-PDO *filled square* and lactic acid *filled triangle*

To investigate the effect of the deletion of *pduC* and *ldhA* on 2,3-BDO production, fed-batch fermentation was conducted with pure glycerol using *K. oxytoca* M3 and the results were compared with the fed-batch fermentation results of the parent strain (*K. oxytoca* M1, Fig. 2a). The fed-batch fermentation was operated over 135 h, but glycerol consumption and 2,3-BDO production were negligible after 114 h. As shown in Table 2, the total amount of glycerol consumed increased in comparison with that of the parent strain (297.1 g/L by *K. oxytoca* M3 vs. 189.5 g/L by *K. oxytoca* M1). The maximum DCW of *K. oxytoca* M3 was also greater than that of *K. oxytoca* M1 (8.6 g/L vs. 4.7 g/L) (Figs. 2a, 4a). These results clearly indicate that the deletion of *pduC* and *ldhA* positively affected glycerol uptake and cell growth. More importantly, the disruption of *ldhA* resulted in a nearly abolished lactic acid production (0.7 g/L, Table 2), and 2,3-BDO production was remarkably increased up to 1.9-fold of the parent strain (59.4 vs. 115.0 g/L). The yield of 2,3-BDO with *K. oxytoca* M3 was also much higher than that of *K. oxytoca* M1 owing to the remarkable reduction of lactic acid production (Table 2). Carbon recovery to 2,3-BDO was 53 % (mole/mole) of the consumed glycerol and the rest of the carbon would be incorporated to CO_2_ (2 mol of CO_2_ emission per mole 2,3-BDO production), cell mass, and by-products (1,3-PDO, lactic acid, ethanol, etc.).

Regarding the formation of by-products, the concentration of ethanol was increased (4.0 vs. 9.3 g/L) (Table 2) by the deletion of *pduC* and *ldhA*, probably because increased NADH availability would induce the NADH-consuming ethanol synthesis pathway for NADH/NAD^+^ balance. The production of 1,3-PDO (6.9 g/L) by *K. oxytoca* M3 was lower than that of *K. oxytoca* M1 (8.9 g/L), but it was higher than that of *K. oxytoca* M2 (0.8 g/L) (Fig. 3). This result suggests that an alternative glycerol dehydratase other than PduC might be involved in the production of 1,3-PDO to maintain NADH/NAD^+^ balance in *K. oxytoca* M3. However, considering that the concentration ratio of 1,3-PDO to 2,3-BDO obtained with *K. oxytoca* M3 was much lower than that obtained with *K. oxytoca* M1 (0.06:1 vs. 0.15:1 in 1,3-PDO:2,3-BDO ratio), the deletion of *pduC* was still effective to decrease carbon flux to 1,3-PDO.

It has been known that a mixture of meso-2,3-BDO (consuming 1 mol of NADH per mole of 2,3-BDO production) and (2S,3S)-2,3-BDO (consuming 2 mol of NADH per mole of 2,3-BDO production) is produced by *K. oxytoca* [2] (Fig. 1). Because the excess NADH due to the significantly deceased NADH-requiring metabolite production (lactic acid and 1,3-PDO) should be consumed to maintain the redox balance, we analyzed the ratio of (2S,3S)-2,3-BDO to meso-2,3-BDO. Interestingly, the ratio of (2S,3S)-2,3-BDO to meso-2,3-BDO for *K. oxytoca* M3 was higher than *K. oxytoca* M1 (1.04:1 vs. 0.79:1), implying that *K. oxytoca* M3 consumed more NADH than *K. oxytoca* M1 per mole of 2,3-BDO production (1.51 vs. 1.44 mol of NADH). In fact, assuming that the aerobic route was involved to convert glycerol to pyruvate (i.e., 1 mol of NADH generation per mole of glycerol) (Fig. 1), the NADH recovery based on the total consumed NADH for metabolite production (2,3-BDO, 1,3-PDO, lactic acid, and ethanol) and total NADH generation from glycerol were similar for *K. oxytoca* M3 and *K. oxytoca* M1 (77 vs. 75 %). However, the percentage of NADH consumption toward 2,3-BDO production out of total NADH consumption in *K. oxytoca* M3 was much higher than in *K. oxytoca* M1 (accounting for 79 and 65 % of total NADH consumption, respectively). This higher NADH consumption due to the higher (2S,3S)-2,3-BDO portion by *K. oxytoca* M3 would explain how the redox balance was maintained despite the deletion of *pduC* and *ldhA.*

In the meantime, we found that the ratio of (2S,3S)-2,3-BDO to meso-2,3-BDO produced by *K. oxytoca* M1 was 0.11:1 and 1.06:1 from glucose and glycerol, respectively, in flask cultures (Additional file 1: Fig. S3). When *K. oxytoca* KCTC1686 (99.5 % 16S rDNA sequence similarity to *K. oxytoca* M1) [26] was tested, the ratio of (2S,3S)-2,3-BDO to meso-2,3-BDO produced was 0.11:1 and 0.44:1 from glucose and glycerol, respectively. Therefore, the high production of 2,3-BDO by *K. oxytoca* M1 and *K. oxytoca* M3 from glycerol compared to other *Klebsiella* strains might be partially attributed to the regulation of redox balance by significantly increasing the (2S,3S)-2,3-BDO portion.

### High production of 2,3-BDO using crude glycerol by *K. oxytoca* M3 in fed-batch fermentation

As seen in Fig. 2, crude glycerol was more effective in 2,3-BDO production by *K. oxytoca* M1. Therefore, fed-batch fermentation using crude glycerol was carried out with *K. oxytoca* M3 to investigate whether a higher 2,3-BDO production would be achieved in comparison to the results in Fig. 4a.

Unlike 2,3-BDO production from pure glycerol by *K. oxytoca* M3, which stopped after 114 h, 2,3-BDO production from crude glycerol occurred until 156 h. When crude glycerol was used as the sole carbon source for *K. oxytoca* M3, DCW was much higher than those obtained with pure glycerol (Table 2). Furthermore, 2,3-BDO concentration (131.5 g/L) and yield (0.44 g/g) were higher than those obtained with pure glycerol by 1.13-fold and 1.14-fold, respectively. The 2,3-BDO productivity obtained using crude glycerol was similar to that obtained with pure glycerol during about 100 h of fermentation (1.10–1.15 g/L/h), but decreased to 0.84 g/L/h as the fermentation was prolonged up to 156 h.

As expected, only a small amount of lactic acid (0.8 g/L) was produced. Notably, in contrast to the fed-batch fermentation using pure glycerol by *K. oxytoca* M3 (Fig. 4a), 1,3-PDO was not detected during 156 h of fed-batch fermentation using crude glycerol (Fig. 4b). Moreover, ethanol production (1.9 g/L) from crude glycerol was much lower than that from pure glycerol (9.3 g/L) (Table 2). This decrease of net NADH-consuming by-product formation (1,3-PDO and ethanol, Additional file 1: Table S1) under aerobic condition was not likely attributed to the increase of NADH consumption toward 2,3-BDO production, because the ratio of (2S,3S)-2,3-BDO to meso-2,3-BDO (0.92:1) using crude glycerol was slightly lower than that using pure glycerol (1.04:1). It is not clear why crude glycerol is more effective than pure glycerol in 2,3-BDO production with less formation of by-product. A possible reason would be the presence of nutrients stimulating cell growth. Considering the enhanced cell growth with crude glycerol, the regeneration of NAD^+^ via electron transport chain under aerobic conditions might be increased to generate more energy (i.e., ATP). Because of this additional NADH consumption, the production of net NADH-consuming by-product might be decreased. Further investigation would be needed to elucidate the effect of crude glycerol on metabolism.

Table 4 compares the 2,3-BDO production from glycerol as the sole carbon source or the mixture of glycerol and sugar provided as a co-substrate from previous reports and this study. Until now, Petrov and Petrova [21] reported the highest production of 2,3-BDO (70 g/L) by *K. pneumoniae* G31 using pure glycerol as the sole carbon source with a yield of 0.39 g/g and productivity of 0.47 g/L/h through the application of forced pH fluctuation. Yang et al. [25] obtained a high concentration (83.3 g/L), yield (0.42 g/g) and productivity (0.87 g/l/h) of 2,3-BDO by *B. amyloliquefaciens* using beet molasses as a co-substrate in addition to biodiesel-derived glycerol; however, using crude glycerol as the sole carbon source, *B. amyloliquefaciens* produced only 43.1 g/L of 2,3-BDO with a yield of 0.38 g/g and productivity of 0.45 g/L/h. In this study, the efficient 2,3-BDO production from biodiesel-derived glycerol was fulfilled by the deletion of the *pduC* and *ldhA* genes in *K. oxytoca* M3 to reduce the formation of 1,3-PDO and lactic acid, and consequently the carbon flux was mainly redirected to 2,3-BDO. More importantly, use of the double deletion mutant and crude glycerol resulted in 1,3-PDO-free 2,3-BDO production, involving no concern to separate 1,3-PDO from 2,3-BDO-containing cell broth.

**Table 4:** Comparison of 2,3-BDO production from glycerol

Host bacteria	Carbon sources	Culture mode	Concentration (g/L)		2,3-BDO productivity (g/LH)	2,3-BDO yield (g/g)	References
			2,3-BDO	1,3-PDO			
*K. pneumoniae*	Pure glycerol	Fed batch	49.2	10.6	0.18	0.36	[20]
*K. pneumoniae*	Pure glycerol	Fed batch	70	16.3	0.47	0.39	[21]
*B. amyloliquefaciens*	Crude glycerol	Fed batch	43.1	–	0.45	0.38	[25]
*B. amyloliquefaciens*	Crude glycerol + molasses	Fed batch	83.3	–	0.87	0.42	[25]
*K. oxytoca*	Crude glycerol	Batch flask	4.8	8.4		0.14	[19]
*K. oxytoca*	Crude glycerol + glucose	Batch flask	8.0	3.8		0.22	[19]
*K. oxytoca* M3	Pure glycerol	Fed batch	115.0	6.9	1.01	0.39	This study
*K. oxytoca* M3	Crude glycerol	Fed batch	131.5	0	0.84	0.44	This study

Overall, a new record of the highest 2,3-BDO concentration from crude glycerol (131.5 g/L) as the sole carbon source was achieved with the highest productivity (0.84 g/L/h) and yield (0.44 g/g crude glycerol) without 1,3-PDO production. It is worth noting that 2,3-BDO concentration obtained using crude glycerol in this study is comparable to the 2,3-BDO production from glucose reported by Ji et al. (130 g/L) and Park et al. (113 g/L) using *K. oxytoca* strains [6, 8]. Further improvement in 2,3-BDO productivity would make it more feasible to produce 2,3-BDO from biodiesel-derived glycerol for industrial use.

## Conclusions

In this study, biodiesel-derived glycerol was used as the sole carbon source for 2,3-BDO production by the engineered strain *K. oxytoca* M3. Enhanced 2,3-BDO production from crude glycerol was achieved by disruption of the *pduC* and *ldhA* genes, which resulted in a nearly abolished lactic acid and 1,3-PDO production. To the best of our knowledge, 2,3-BDO concentration (131.5 g/L), productivity (0.84 g/L/h), and yield (0.44 g/g) achieved in this study are the highest levels in glycerol-based 2,3-BDO production reported to date, demonstrating that biodiesel-derived glycerol could be used to produce 2,3-BDO cost-effectively by the metabolically engineered strain *K. oxytoca* M3.

## Methods

### Microorganisms and media

All bacterial strains and plasmids used in this study are listed in Table 3. *K. oxytoca* M1 was deposited in the Korean Culture Center of Microorganisms (KCCM) as KCCM 1177P. *K. oxytoca* KCTC1686 (equivalent to ATCC8724) was purchased from the Korean

Collection for type culture (KCTC, Korea). *K. oxytoca* M1 and its mutants were pre-cultured in Luria–Bertani (LB) medium at 30 °C. The defined medium used for flask fermentation contained (per L of distilled water): K_2_HPO_4_ 13.7 g, KH_2_PO_4_ 2 g, (NH_4_)_2_HPO_4_ 3.3 g, (NH_4_)_2_SO_4_ 6.6 g, MgSO_4_·7H_2_O 0.25 g, FeSO_4_·7H_2_O 0.05 g, ZnSO_4_·7H_2_O 0.001 g, MnSO_4_·H_2_O 0.01 g, CaCl_2_·2H_2_O 0.01 g, and EDTA 0.05 g. Pure glycerol or crude glycerol were added to the defined medium as needed. Crude glycerol provided by GS Caltex Corporation (South Korea) contained (wt/wt): 81.7 % of glycerol, 10.5 % of water, 5 % of MONG (matter organic non-glycerol), 2.9 % of ash, 2.4 % of sodium, and less than 0.01 % of methanol, magnesium and potassium. The medium used for pH-controlled fermentation was the defined medium supplemented with 5 g/L yeast extract and 10 g/L casamino acid [16].

### Fermentation procedures (flask and fermentor)

For flask fermentation without pH control, pre-culture cultivation was carried out overnight in LB medium at 30 °C and 200 rpm. Then, the seed culture was inoculated in a 100 mL Erlenmeyer flask (5 %, v/v) containing 20 mL of the defined medium. Pure glycerol and crude glycerol (35–40 g/L) were added to the defined medium to investigate metabolite production patterns (initial pH 7.0, 30 °C, 200 rpm, 48 h). All flask experiments were performed in triplicate.

All trials for 2,3-BDO fermentation with pH control in this study were conducted in a 3 L stirred fermenter (Fermentec FMT ST, South Korea) with a working volume of 1 L. For seed culture preparation, *K. oxytoca* M1 and the derivatives were inoculated into 100 mL of LB medium and cultivated on a rotary shaker at 200 rpm at 30 °C for 10 h. The seed culture (10 %, v/v) was then inoculated into the defined medium supplemented with 10 g/L casamino acid and 5 g/L yeast extract. All cultivations were carried out at 30 °C, and the pH level was maintained at 6 by automatic addition of 5 N NaOH. The aeration rate was controlled at 1.0 volume of air per volume of liquid per minute (vvm) with the agitation speed of 400 rpm.

The batch cultivation was carried out at 30 °C using fermentation medium containing 90 g/L of glycerol. Fed-batch fermentation was carried out with an initial glycerol concentration of 90–100 g/L, and then a concentrated solution containing 800 g/L of pure glycerol or crude glycerol was fed into the fermenter as required.

### Construction of the *pduC* deletion mutant

The *pduC* gene (accession number AIE72369) deletion mutant, *K. oxytoca* M2, was developed from *K. oxytoca* M1 using the λ Red recombination method [30, 31] (Additional file 1: Fig. S1A). The full genome sequence of *K. oxytoca* M1 (CP008841) was provided by Macrogen Inc. (Seoul, South Korea) [32]. Two PCR products including the upstream and downstream regions of *pduC* in genomic DNA were generated using the primers pduUp1 and pduUp2 to get 1031 bp of fragments and using the primers pduDown1 and pduDown2 to obtain 843 bp of fragments, respectively (Table 5). In addition, PCR using the pTOP-FCF plasmid as a template was performed with the primers pduCUDFCF1 and pduCUDFCF2 (Table 5) to get 930 bp of the PCR products (step 1 in Additional file 1: Fig. S1A). The 2639 bp fragments spanning the upstream regions (1031 bp), FRT-Cm^R^-FRT (930 bp), and downstream regions (843 bp) were amplified by the primers pduCDown2 and pduCUP1, using PCR products as a template by the overlap extension PCR method.

**Table 5:** Oligonucleotides used in this study

Oligonucleotides	Sequence^a^	Source
pduCUp1	5′-TTATGCTTCTTTTTTACGCAGCTTATCG-3′	This study
pduCUp2	5′-TTTCTAGAGAATAGGAACTTC GGGGAACTGCATCATGGAA-3′	This study
pduCUDFCF1	5′-TTCCATGATGCAGTTCCCC GAAGTTCCTATTCTCTAGAAA-3′	This study
pduCUDFCF2	5′-GGCACAATTTTTTAATCTTA GAAGTTCCTATACTTTCTAGA-3′	This study
pduCDown1	5′-TCTAGAAAGTATAGGAACTTC TAAGATTAAAAAATTGTGCC-3′	This study
pduCDown2	5′-AATAAGCCTCAGAAAATTGAGTTA GAAATAAAGTTGA-3′	This study
pduCcon1	5′-CCGTTTTCAACCAGCGTCAG-3′	This study
pduCcon2	5′-CCGGTAATTCTCACCCGGAG-3′	This study
ldhADown1	5′-TTACCAGACCACGGATTGCG-3′	This study
ldhADown2	5′-CTTTCTAGAGAATAGGAACTTC CTTTCCCTTTTGTGCTCCT-3′	This study
ldhAUDFCF1	5′-GGAGCACAAAAGGGAAAG GAAGTTCCTATTCTCTAGAAAG-3′	This study
ldhAUDFCF2	5′-TATTATCACTGGAGAAAAGTCTT GAAGTTCCTATACTTTCTAGAGAA-3′	This study
ldhAUP1	5′-TTCTCTAGAAAGTATAGGAACTTC AAGACTTTTCTCCAGTGATAATA-3′	This study
ldhAUP2	5′-GCGGGCTTTCATTGAGTGAG-3′	This study
ldhAcon1	5′-GGGAATTGTAACTTTATCGCAGGC-3′	This study
ldhAcon2	5′-CCGTGACGGTATTATCACTGGA-3′	This study

^a^Underlined sequences are homologous with the FRT region

Then, the λ Red recombinase expression plasmid pRedET was transformed into *K. oxytoca* M1 by electroporation at 12.5 kV/cm, 200 Ω, 25 μF (Gene PulserXcell, Biorad). The 2639 bp linear fragments containing Cm^R^ were transformed to *K. oxytoca* M1 haboring pRedET, and the chloramphenicol-resistant colonies were selected on LB agar plates containing 25 μg/mL of chloramphenicol at 37 °C (step 2 and step 3 in Additional file 1: Fig. S1A). Deletion of the *pduC* gene from the chromosome was confirmed by the size of the PCR product (1010 bp) obtained using the pduCcon1 and pduCcon2 primer pair (step 3 in Additional file 1: Fig. S1A, Table 5). To remove the Cm^R^ cassette from the chromosome, FLP expression plasmids (707-FLPe, Gene Brideges, Germany) were transformed into the cells and the tetracycline-resistant transformants were selected at 30 °C. After cultivation at 42 °C overnight, the desirable antibiotic-susceptible transformants which lost the FRT flanking Cm^R^ gene and the 707-FLPe were selected. The resulting mutant was confirmed by PCR (153 bp, step 4 in Additional file 1: Fig. S1A) using the pduCcon1 and pduCcon2 primer pair (Table 5).

### The *pduC* and *ldhA* double deletion mutant

The *pduC* and *ldhA* (encoding lactate dehydrogenase, accession number AIE70186) double deletion mutant, *K. oxytoca* M3, was constructed from *K. oxytoca* M2 (*K. oxytoca* M Δ*pduC*) using the λ Red recombination method as described above. Two PCR products including the upstream and downstream regions of *ldhA* in genomic DNA were generated by using the primers ldhAUp1 and ldhAUp2 (Table 5) to get 993 bp of PCR products and by using the primers ldhADown1 and ldhADown2 (Table 5) to obtain 1091 bp of PCR products (step 1 in Additional file 1: Fig. S1B), respectively. In addition, the 932 bp of PCR products were generated by using primers (ldhAUDFCF1 and ldhAUDFCF2) that included homology extensions to the upstream and downstream regions and priming sequences for pTOP-FCF as a template (Table 5). The 2665 bp fragments were amplified by the primer pair, ldhADown1 and ldhAUP2, using PCR products (993, 932, and 1091 bp) as a template by the overlap extension PCR method.

After construction of linear fragments for homologous recombination of the *ldhA* gene, the next process was conducted in the same manner as the *pduC* deletion mutant construction process (step 2 and 3 in Additional file 1: Fig. S1B). Deletion of the *ldhA* gene from the chromosome was confirmed by PCR (150 bp, step 4 in Additional file 1: Fig. S1B) using the ldhAcon1 and ldhAcon2 primer pair (Table 5).

### Analytical methods

Dry cell weight (DCW, g/L) was calculated from optical density at 600 nm (OD_600_) using the calibration curve of OD_600_ and the dry cell weight of *K. oxytoca* M1. The OD_600_ of the broth was measured by UV–visible spectrophotometry (Cary 60 UV–Vis, Agilent Technologies, USA) with appropriate dilution.

The concentrations of meso-2,3-BDO, (2S,3S)-2,3-BDO, (2R,3R)-2,3-BDO, 1,3-PDO, and ethanol were measured using a gas chromatograph (Shimadzu GC-2010, Kyoto, Japan) equipped with a flame ionized detector (FID) and an HP-Chiral-20B GC column (30 m X 0.32 mm X 0.25 μm) (Agilent) under the conditions described previously [26, 27]. Glycerol and lactic acid were analyzed using a high-performance liquid chromatograph (HPLC) (Agilent 1260, Waldbronn, Germany) equipped with a refractive index detector (RID) and an Aminex HPX-87 H Ion Exclusion Column (300 × 7.8 mm, Bio-Rad, Hercules, CA, USA) under the following conditions: sample volume of 20 μL, mobile phase of 5 mM H_2_SO_4_, flow rate of 0.5 mL/min, and column temperature of 50 °C. All culture samples tested were preliminarily filtered through a membrane filter (0.45 μm pore size, Millipore, USA).

## Additional file

**Additional file 1: Figure S1.** Schematic representation for generating of deletion mutants using the *λ* Red recombination method. **Figure S2**. PCR verification for the deletion of *pduC* and *ldhA* genes from *K. oxytoca* M1 and *K. oxytoca* M2, respectively. **Figure S3**. Relative production of meso-2,3-BDO and (2S, 3S)-2,3-BDO by *K. oxytoca* M1and *K. oxytoca* KCTC1686 using glucose or glycerol as the carbon sources. **Table S1**. Net NADH balance for the corresponding product formation per mole of glycerol.

## Abbreviations

2,3-BDO: 2,3-butanediol
1,3-PDO: 1,3-propanediol
PduC: glycerol dehydratase large subunit
LDH: lactate dehydrogenase
DCW: dry cell weight
bp: base pair
PCR: polymerase chain reaction
NADH: nicotinamide adenine dinucleotide plus hydrogen
OD_600_: Optical density at 600 nm
