# Supplementary material for: High production of 2,3-butanediol from biodiesel-derived crude glycerol by metabolically engineered Klebsiella oxytoca M1
Source: Biotechnol Biofuels. 2015 Sep 15;8:146. doi: 10.1186/s13068-015-0336-6 (PMC4570460; doi:10.1186/s13068-015-0336-6)
Supplement: Supplementary file 1 — Additional file 1: Figure S1. Schematic representation for generating of deletion mutants using the λ Red recombination method. Figure S2. PCR verification for the deletion of pduC and ldhA genes from K. oxytoca M1 and K. oxytoca M2, respectively. Figure S3. Relative production of meso-2,3-BDO and (2S, 3S)-2,3-BDO by K. oxytoca M1and K. oxytoca KCTC1686 using glucose or glycerol as the carbon sources. Table S1. Net NADH balance for the corresponding product formation per mole of glycerol. [file 13068_2015_336_MOESM1_ESM.pdf]

Supporting Information

Figure S1

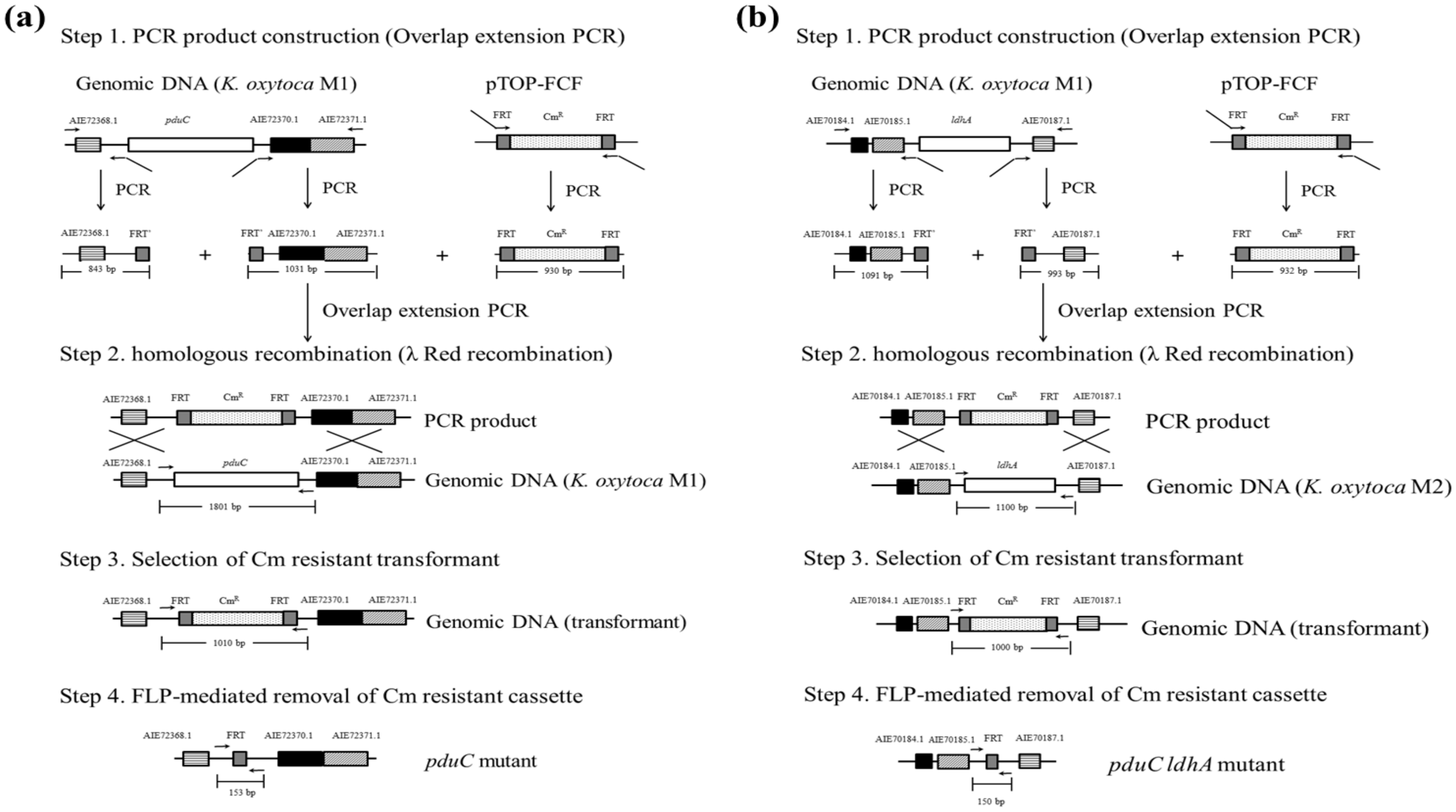

Figure S1. Schematic representation for generating of deletion mutants using the λ Red recombination method.

(a) Deletion of the glycerol dehydratase large subunit (*pduC*) gene from *K. oxytoca* M1. By transforming 2639 bp of linear fragment to *K. oxytoca* M1 as described in the Material and Methods section, the deletion mutant with the Cm^R gene was confirmed by colony PCR using locus specific primers binding the flanking region of *pduC* (pduCcon1 and pduCcon2) (Table 3). The PCR products of 1010 bp (Step 3) were detected from the isolated chloramphenicol resistant colonies (wild type: 1801 bp), demonstrating the replacement of the *pduC* gene with the FRT-flanking Cm^R cassette. Upon elimination of the Cm^R cassette by transformation of the FLPe expressing plasmid 707-FLPe, the resulting mutant gave the expected size of the PCR fragment (153 bp) with the same locus specific primers (pduCcon1 and pduCcon2) (Fig. S2, lane 3). The fragment of 1801 bp (Fig. S2, lane 2) containing intact *pduC* was amplified from the wild-type strain.

(b) Deletion of the lactate dehydrogenase (*ldhA*) gene from *K. oxytoca* M2. Similar scheme used for *K. oxytoca* M2 construction was applied with the specific primers (Table 3) as described in Material and Methods section.

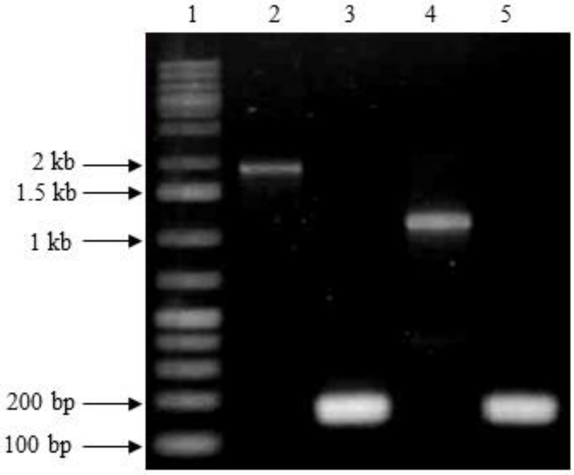

Figure S2. PCR verification for the deletion of *pduC* and *ldhA* genes from *K. oxytoca* M1 and *K. oxytoca* M2, respectively. Lane 1, molecular weight marker; Lane 2, PCR product of wild type strain with pduCcon1, 2 primer pair (1801 bp); Lane 3, PCR product of *pduC* deletion mutant strain with pduCcon1, 2 primer pair (153 bp); Lane 4, PCR product of wild type strain with ldhAcon1, 2 primer pair (1100 bp); Lane 5, PCR product of *pduC* and *ldhA* double deletion mutant with ldhAcon1, 2 primer pair (150 bp).

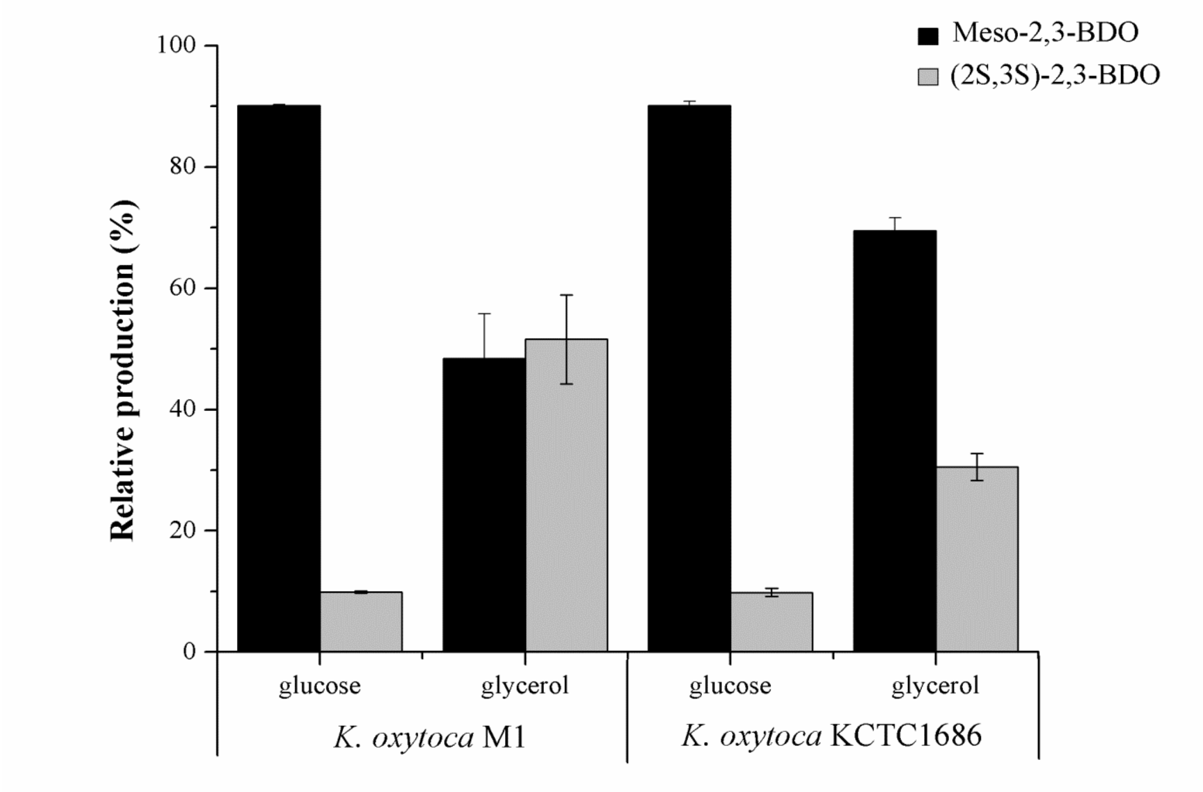

Figure S3. Relative production of meso-2,3-BDO and (2S, 3S)-2,3-BDO by *K. oxytoca* M1and *K. oxytoca* KCTC1686 using glucose or glycerol as the carbon sources. Fermentation was carried out in 100 mL flask containing 20 mL defined medium with 40 g/L of carbon sources at 200 rpm and 30 ˚C. Relative production was calculated as the percentage of each 2,3-BDO isomer out of the total 2,3-BDO production.

Table S1. Net NADH balance for the corresponding product formation per mole of glycerol

Product	mole	Aerobic pathway		
		Generated	Consumed	Net
Meso-2,3-BDO	0.5	+1 NADH	-0.5 NADH	0.5
(2S, 3S)-2,3-BDO	0.5	+1 NADH	-1 NADH	0
Ethanol	1	+1 NADH	-2 NADH	-1
Lactic acid	1	+1 NADH	-1 NADH	0
		Reductive pathway		
1,3-PD	1	0	-1 NADH	-1
